# Design and Development of Lubricating Material Database and Research on Performance Prediction Method of Machine Learning

**DOI:** 10.1038/s41598-019-56776-2

**Published:** 2019-12-30

**Authors:** Dan Jia, Haitao Duan, Shengpeng Zhan, Yongliang Jin, Bingxue Cheng, Jian Li

**Affiliations:** 1 State Key Laboratory of Special Surface Protection Materials and Application Technology, Wuhan Research Institute of Materials Protection, Wuhan, China; 2 State Key Laboratory of Tribology Tsinghua University, Tsinghua University, Beijing, China

**Keywords:** Materials science, Physics

## Abstract

Long developing period and cumbersome evaluation for the lubricating materials performance seriously jeopardize the successful development and application of any database system in tribological field. Such major setback can be solved effectively by implementing approaches with high throughput calculation. However, it often involves with vast number of output files, which are computed on the basis of first principle computation, having different data format from that of their experimental counterparts. Commonly, the input, storage and management of first principle calculation files and their individually test counterparts, implementing fast query and display in the database, adding to the use of physical parameters, as predicted with the performance estimated by first principle approach, may solve such setbacks. Investigation is thus performed for establishing database website specifically for lubricating materials, which satisfies both data: (i) as calculated on the basis of first principles and (ii) as obtained by practical experiment. It further explores preliminarily the likely relationship between calculated physical parameters of lubricating oil and its respectively tribological and anti-oxidative performance as predicted by lubricant machine learning model. Success of the method facilitates in instructing the obtainment of optimal design, preparation and application for any new lubricating material so that accomplishment of high performance is possible.

## Introduction

Lubrication materials are the essentially key materials to improve the functionality of new energy vehicles, aerospace, marine ships and intelligent machineries^1–3^, and their further development. Much of research work on problems associated with lubricating materials has been carried out, which results in abundant simulation or test data^4–7^. However, these individual data are currently still not being effectively utilized, and difficulty in evaluating performance of lubricating materials in various industrial applications is still widely remaining as a major problem. A way to relieve such problem may be combining technique of first-principles high-throughput calculation with the use of database embedding massive research information and analytical tools. This, integrating with the possible component of screening or searching, according to specific needs, gears the trend in developing and/or exploring new materials^8^. Commonly, the particularity of analog computation often results in problematic files of data structure, and data storage, and its processing of data calculated by first-principles, which are barriers of database platform and such problem requires to break through effectively.

Establishment of an effective and efficient data structure generally requires the premise for building up an appropriate database platform. This, in turn, inevitably affects the structure and techniques on data storage, data retrieval, and machine learning^9^. In practically operational process, it often needs to adjust/compromise the logical and physical relationship between the real time data. Typically, most simulated data are just stored in local hard disks and likely to be erased carelessly. Hence, choosing some storage mode to give a long-term and secure storing is thus necessary and recommended. Establishment of a database platform not only can solve the problem of massive simulated data generated by calculation, it also helps to standardize and/or integrate the simulated and tested data so as to improve the effective integration and utilization of the data.

Input and output to these files of first principles usually contain large amount of vital information and rather complex contents. Major information like energy values, fermi energy values and eigenvalues must be suitably and efficiently extracted and re-stored for further viewing, Furthermore, feasibility in displaying information such as band structure and spectrum often varies with the capacity of the processing graphics software. Therefore, output visualization is also one of the problems to be solved in the procedure of establishing database architecture.

Aiming at solving the problems as stipulated above, some researchers have accomplished partly the work in constructing of First Principle database: typically, AFOW^10^, Materials Project^11^, MedeA^12^, MatCloud^13^, NOMAD^14^ and the software associated with Materials Informatics Platform (MIP)^15^. Among these, the first four platforms are known as high-throughput platforms built with the first-principle VASP software packages, which are capable providing data to be shared between computing platforms and calculation results. The software is mainly embedded with inorganic crystal structure data, and it has some constraint on the selection of first-principles calculation software, moreover, lacks the support of experimental data. Although NOMAD is equipped with the supports to the users in uploading and downloading input and output files for various computing software processes, its data analysis is not powerful enough. However, Materials Informatics Platform is generally considered as a computing platform for thermoelectric materials, a series of consultations suggests that its database platforms are lacking of lubrication data of material and its simulated data are unable to combine with their experimental counterparts. That is, there does not solve the problems of diverse/sparse data storage and information extraction. It also needs conducting associated theoretical study to correlate the calculated physical parameters to the properties of materials. Hence, predictive research on material performance becomes critical in accelerating its development. Such realization resulted in the launch of Material Genome Project in the United States in 2011. As the research methods involve with high throughput experiments, high performance computing and data depth analysis, it has drawn many scientific researchers and substantial resources into the project, specifically working in exploring the method of data mining and performance prediction^16–19^. Its nature of massive data accumulation, combining with material performance prediction, has led to many new materials to be discovered and developed.

In view of the above mentioned, this paper specifically utilizes Window system to construct an integrated development environment of Hypertext Preprocessor (abbreviated as: PHP)/MySQL and PHP/MongoDB. To accomplish such purpose, a Structure-Performance database of lubricating materials has been built to store data and analyze the information achievable from high throughput calculation and experiment. The functionalities of the preprocessor and the database fundamentally consist of: uploading and inputting data (both tested and simulated ones) in different formats; searching information by simply keying in keywords or parameters; predicting performance via first principle calculations. By adequately studying the relationship of the calculated parameters and their corresponding performance of lubricating materials, it allows the establishment a machine learning prediction model preliminarily for tribological and anti-oxidative properties of the lubricating oils so that relatively more accurate and meaningful prediction can be achievable.

## Architecture of Lubricating Materials Database

### Construction of integrated development environment for high-throughput computing

**Figure 1:**
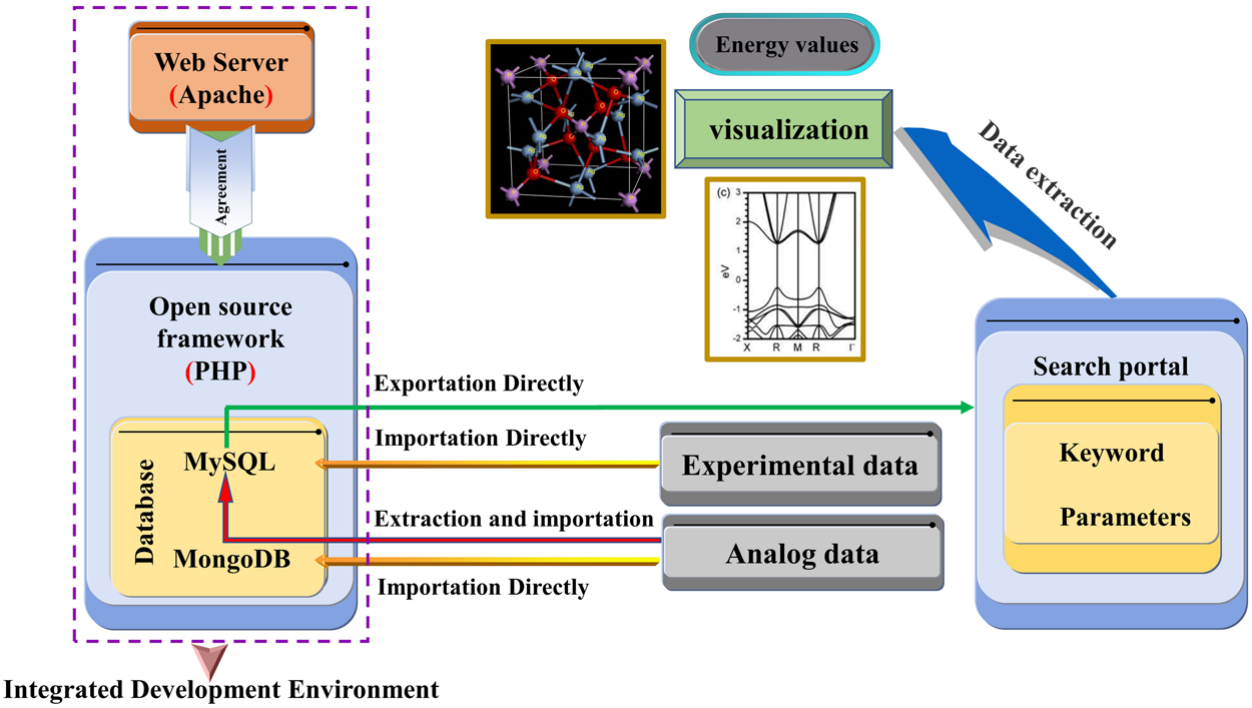
The integrated development environment of database.

For the data processing requirements of high throughput computing and research and development of lubricants, a stable integrated development environment for PHP based on Apache service protocol under Windows environment (Fig. 1) has thus been built. MySQL (relational database) and MongoDB (non-relational database) are generally considered as the integrated environmental platform. In addition, the communication between (i) PHP and MySQL database, and between (ii) PHP and MongoDB database can be realized by suitably configuring. The dynamic link of such two databases often results in a variety of storage forms on specifically importing the test data and the high-throughput calculation results, which commonly has inclination to retain their individually original data^20^. Furthermore, the conversion formats between different data often facilitate the retrieval of these data.

### Construction of front-end and back-end system framework for database website

#### Backstage management system

The background management system is a prerequisite module of database and has great versatility for its internal users. In this design, the background management system remains the same interface as the front-end system interface. Owing to the needs in development stage, background management system is effectively categorized into three types, which are typically as data management, user management and system management. Such data management system is mainly used in managing data and users, such as: data deletion, user addition and deletion, role management, setting user permissions, and so on.

#### Front-end application system

**Figure 2:**
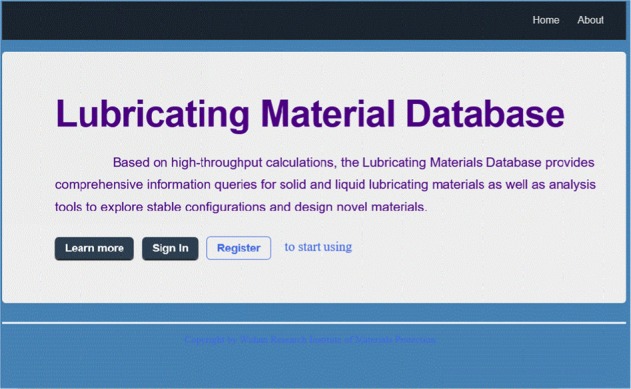
The main front-end interface.

The pages of front-end management are in fact interactive interface mainly gearing the communication between users and personalized according to their individually analytical levels. Their typical welcome interface of a database website is shown in Fig. 2, which gives a brief illustration of a website, and its login page and registration page for its users. Protection for user login and registration can be realized by the security functional block in the website data. Moreover, users are restrained to log in repeatedly so as to enhance resource saving and to improve the accessing speed.

### Construction of functional module system

#### Logical relationship of functional module system

**Figure 3:**
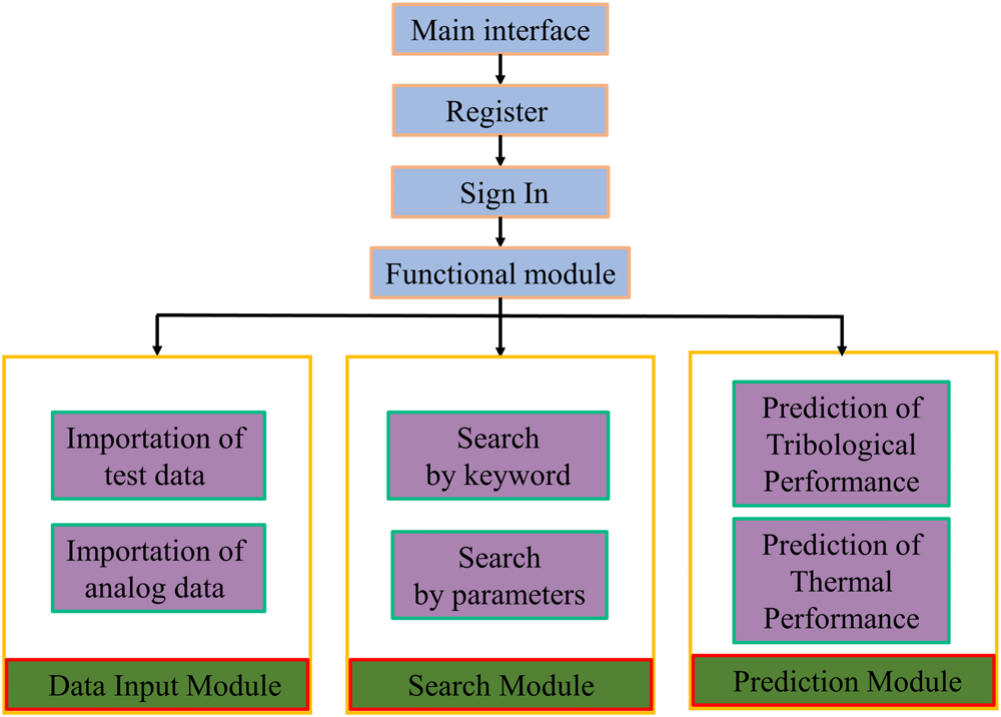
The structure layer and function module.

The structural layer and functional modules of the system are shown in Fig. 3. Basically, one-to-many tree structure is adopted as the logical structure of data, and in its physical structure like order, connection and index. Structures with distinct layers are also made conducive so that realization of design schemes is possible. As seen in Fig. 3, its functional modules are fundamentally divided into three parts, which typically consist of: (i) Data input module, (ii) Search module, and (iii) Prediction module.

#### Relationship schema/ entity-relationship for the MySQL database

**Figure 4:**
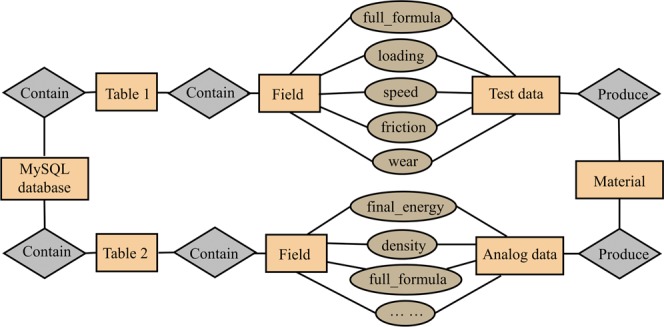
Relationship schema/entity-relationship diagram for the MySQL database.

The MySQL database is established with (i) table names, (ii) table Structure, (iii) fields, (iv) field types, and (v) so on. Furthermore, the optimization of database is also very important for its acceptability to users. When the structure of MySQL is not well designed, its efficiency in developing an encoding process is significantly jeopardized. Figure 4 illustrates the relationship schema/entity-relationship diagram and the interconnection of individual components within this proposed MySQL database.

#### Implementation scheme of functional module system

**Figure 5:**
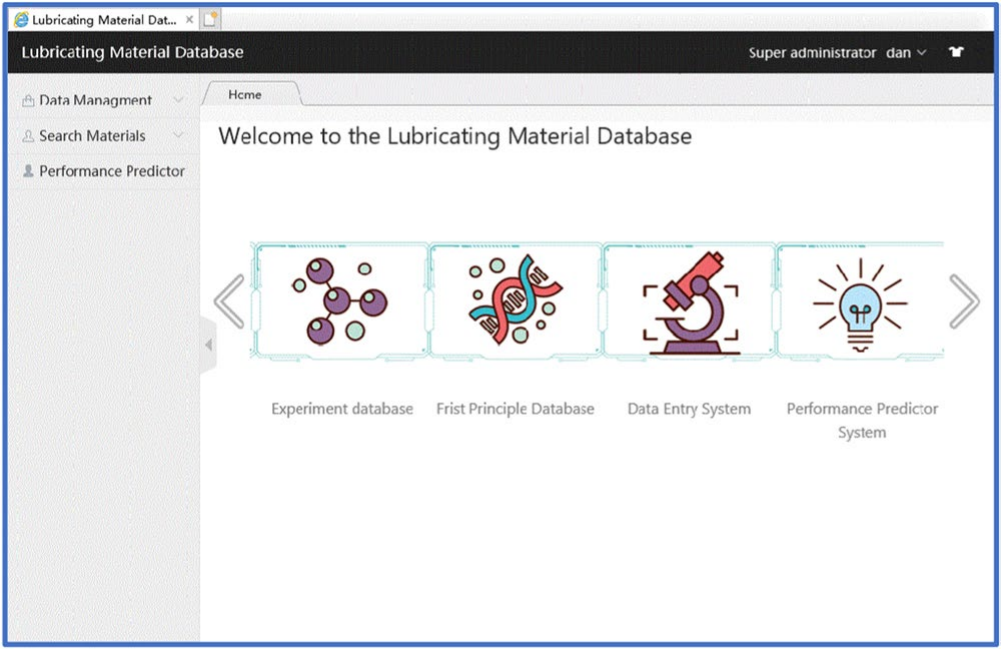
The interface of function module of lubrication material database.

Figure 5 shows the interfacial connection of a function module in the lubrication material database. The realization of its function of inputting data for both test and analog calculation, together with querying components for (i) keyword and parameter, and (ii) prediction of material properties can be simply achievable by clicking different module boxes in the system. The remaining sections in this paper briefly introduce the specific functions of individual modules.

**Figure 6:**
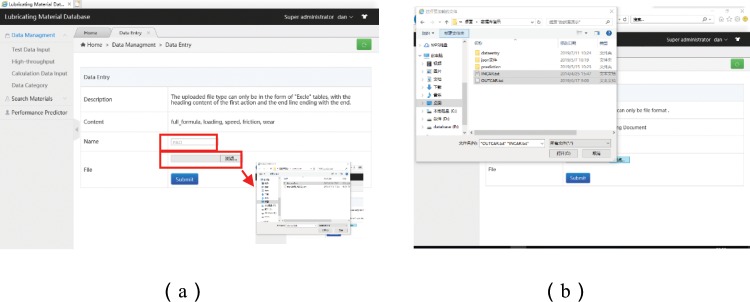
Data entry system of experimental data and analog data.

**Figure 7:**
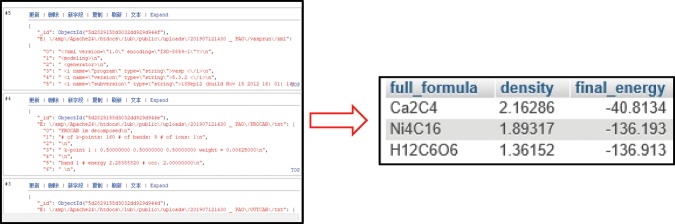
Structural transformation and input of high-throughput computing data.

Data input module: Standardization of data formats is aimed at facilitating data retrieval and improving utilization efficiency. Diversification of operating software is often derived from series of testers and high throughput computing, which recursively leads to type diversification of output files. Data input modules in this paper are specifically designed to include inputting data from both experiments and/or those files calculated by first-principles, which commonly fuse together the values of physical parameter and service performance of the associated materials. As inputting test data is only supported by Excle tables in standard format (Fig. 6(a)), while inputting analog data is supported by TXT documents (Fig. 6(b)), the process of importing data can be routed orderly as follows: (i) entering name of the compound; (ii) selecting upload file from the personal computer, and (iii) clicking “Submit” to complete upload. Thereafter, the system will automatically connect MySQL database and MongoDB database in PC, and it stores test tables in MySQL database, and results of high throughput calculation in MongoDB database, filtering and retrieving key data in the background of the system, and finally by inserting the associated data into the MySQL database (Fig. 7). Its structural transformation mechanism for filtering, extracting and inserting operations basically involves with: (i) converting the obtained string into an array, (ii) selecting the variable and value to be extracted from the array, (iii) re-creating a new array with the variable consistently with the inserted table header, and (iv) updating the variable and value on MySQL database. Effectively sorting and integrating relational and non-relational databases not only meet the requirements for processing special data and for high-throughput computing, it also provides capability for data query and management.

**Figure 8:**
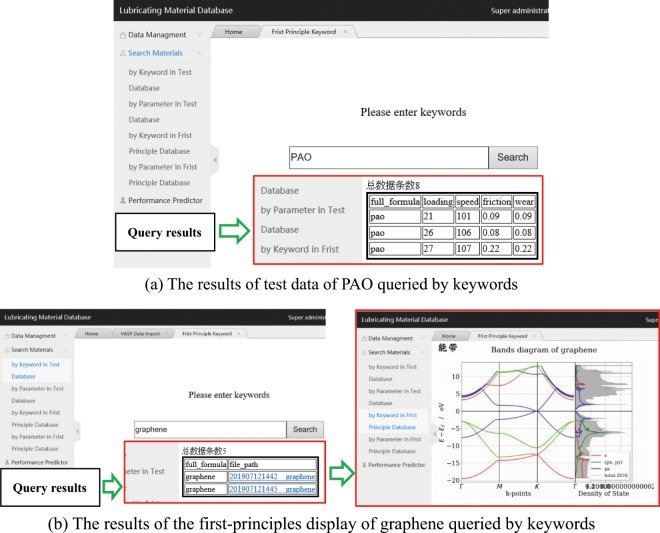
Result display of keyword query.

**Figure 9:**
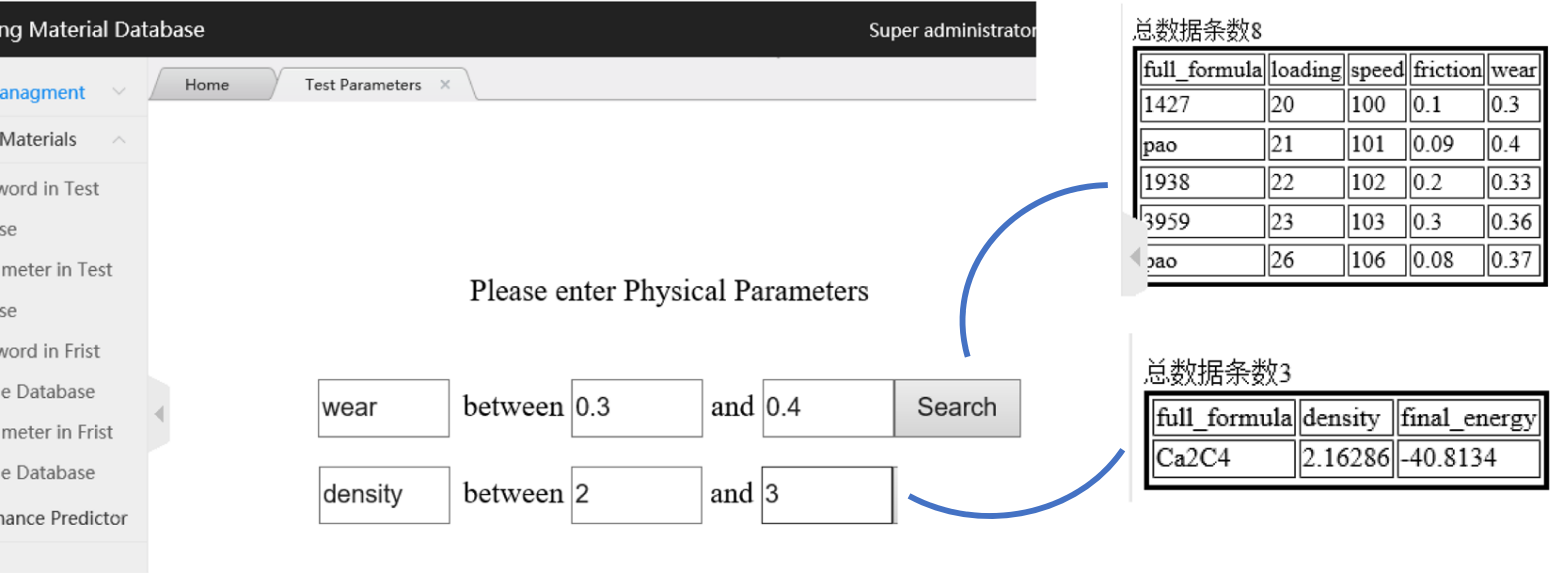
Results display of performance and physical parameters query.

Search module: Query system allows realization of keyword query and physical parameters query, through transmitting the database to SQL statements.The statement of keyword query is: select * from search_keyword where full_formula = ‘$_POST[keyword]; Queries for performance and physical parameters must be implemented in the form of fuzzy queries, such as queries for lubricating materials that conform to a particular range of dipole moments: select * from data entry where $_POST [keyword] between $_POST [a] and $_POST [b]. Example of the results of test data of PAO and first principles display of graphene query by keywords is as illustrated in Fig. 8, whilst Fig. 9 is demonstrating the example in the query results of inputting performance (e.g. wear) or physical parameters (e.g. density), and also their corresponding searching range, which allows the finding of corresponding compounds from its possibly variable.

Prediction module: Series of first principle theoretical methods in predicting material properties are mainly based on Schrodinger equation. Hence, the target of its database construction needs to have capability in screening material and predicting material performance. It is thus crucial to establish a relationship model between parameters and properties using Python language on the basis of data mining technology. Thereafter, an interface connecting Python to PHP has built, and the prediction of performance of lubricating material has been realized by suitably transferring the relational model to the prediction group data. Such prediction system should thus be equipped with capability in predicting the physical and chemical performance, anti-oxidative performance and tribological performance of the associated lubricating materials.

**Figure 10:**
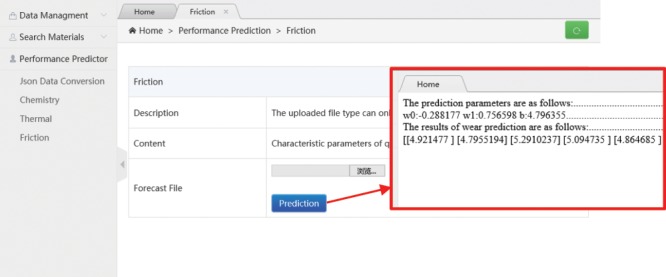
Results display of wear spot diameter prediction.

In the operation of this developed database system, the user is expected importing the results of lubricating material, initially obtained by first-principles calculation, which are followed by clicking “prediction” button to terminate the inputting action. Figure 10 tabulates the predictions of the friction coefficient obtained after introducing the simulation data of the lubricating oil.

## Performance Prediction Method of Lubricating Oil Based on Machine Learning

As material structure always affects its properties and performance, a large number of experiments illustrates that the effects of existence of unsaturated bonds in lubricating oil molecules to its low temperature performance^21–24^. Studies also indicated that length and breakage of carbon chain often change its viscosity and viscosity index, and the branching of ester carbon chain improves its hydrolytic stability. Fracture failure of O-H bond and N-H bond in additives also likely leads to rapid deterioration of lubricating oil performance. In 2016, an article in Nature reported a prediction method specifically focusing on simulated physical characteristics (typically like: charge mobility, photovoltaic characteristics, etc.), which provides guide to synthesis of target materials having specific functions of machine learning^25^. This serves to demonstrate the important role that machine learning plays in discovering the relationship between microstructure and material properties which are normally not revealed in a test process.

Implementation of first principles calculations to software like VASP^26^, Materials Studio^27^ and CASTEP^28^ has been carried out. It has also used Materials Studio simulation software to calculate physical parameters of the lubricating additives in this article, and its outputs are imported into relevant modules in the database, machine learning models within Tensorflow for predicting the associated performance.

### Calculation of quantum mechanics parameters of lubricating oil

**Figure 11:**
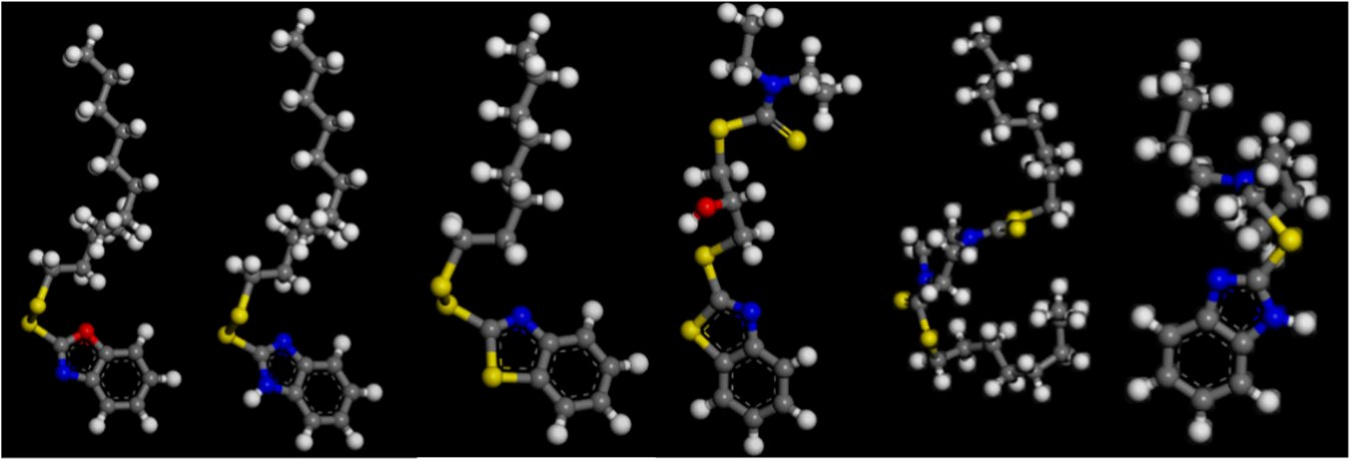
Six molecular structural diagrams of lubricating oil additives.

Molecular model of additives can be established in Materials Studio. However, the establishment process requires the performance of structure optimization of additives by the firstly use of Forcite package^29^ and then DeMol 3 package^30^. After the completion of geometry optimization, it uses Demol 3 package to calculate the anticipated parameters. Figure 11 shows six of these typical molecular models.

Quantum parameters of molecules (such as: molecule surface area, molecule energy, molecule volume, dipole moment, energy orbital, etc.) are subsequently calculateing to the Formula 1, in which X represents the calculated values, Xmin and Xmax represents the minimum and the maximum value in calculations, and Xnorm represents the normalized values. Normally, the normalized parameter values are basically used to build the necessarily relational model.

1 $${\rm{Xnorm}}={\rm{X}}-{\rm{Xmin}}/{\rm{Xmax}}-{\rm{Xmin}}$$

### Construction and importation of machine learning model between feature parameters and wear

**Figure 12:**
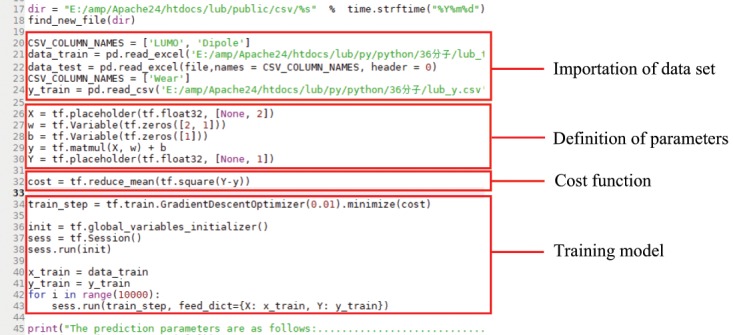
Machine learning model of lubricating oil.

Wear data of the 36 groups of lubricants used for constructing the model in Sec. 3.1 are all from the doctorate thesis of Junyan Zhang^31^. Base oil used in the test is liquid paraffin. Friction and wear tests were carried out on a standard four-ball tester running at a rotational speed of 1450 rpm with test time of 30 min, and its wear scar diameter has been obtained for wear volume estimation^32^. As mentioned previously, a linear regression machine learning model for characteristic parameters (low orbital energy and dipole moment) of lubricating oil and wear has been built by using Tensorflow (Fig. 12). Among the 36 groups, 29 groups are classified as training group and 7 other groups are taken as prediction group. Both training group and other data set are randomly selected from the 36 groups of data, which have been properly numbered. The data imported to training group is by Excle form and with data in the 29 groups, while the other 7 prediction groups are purposefully used for verification there validity.

**Figure 13:**
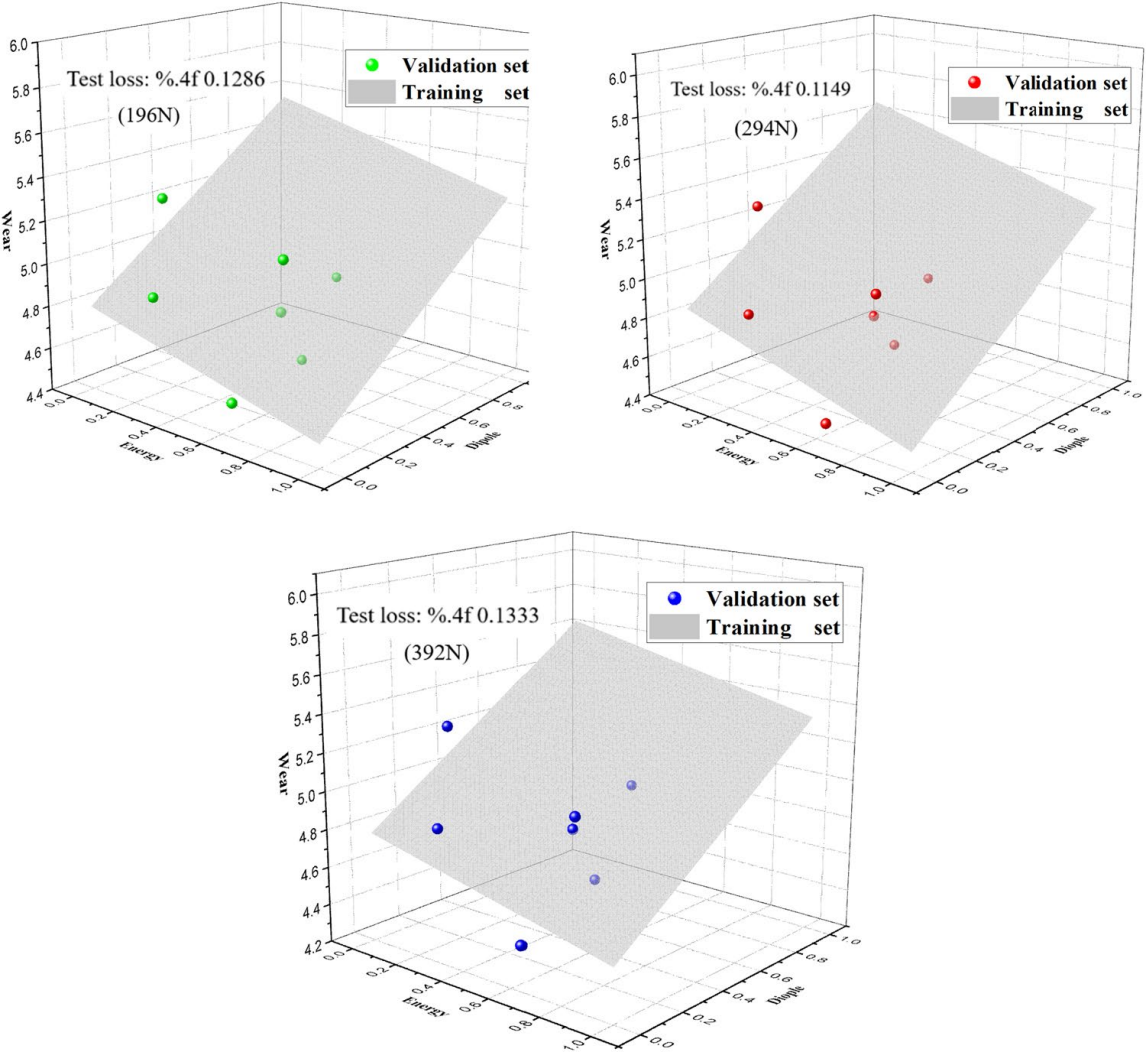
The training and verification loss plots.

The subsequently obtained training model with data from training groups enables defining weights, building cost functions, and adopting gradient descent methods. The model is invoked through the database platform, whilst the prediction counterpart is used to substitute the data from model for predicting wear volume. For facilitating the analysis, comparison of the predicted wear with its actually practical wear, has also been conducted, and their corresponding predicted accuracy is tabulated in Tables 1–3. The loss plot in Fig. 13 compares the discrepancy between training and verification.

**Table 1:** Prediction of wear and comparison with actual wear (load: 196N).

LUMO energy	Total dipole	Prediction values	Actual values	Prediction rate
0.133606	0.216264	4.921477	5.273	93.33%
0.216042	0.081183	4.79552	4.8732	98.41%
0.411698	0.810617	5.291024	4.7826	89.37%
0.454471	0.567473	5.094736	4.4681	85.98%
0.578933	0.31082	4.864686	4.833	99.34%
0.604808	0.040659	4.652826	4.526	97.20%
0.646044	0.24328	4.794245	5.1147	93.73%

**Table 2:** Prediction of wear and comparison with actual wear (load: 294N).

LUMO energy	Total dipole	Prediction values	Actual values	Prediction rate
0.133606	0.216264	4.973328553	5.3326	93.26%
0.216042	0.081183	4.83523244	4.853	99.63%
0.411698	0.810617	5.36052086	4.8413	89.28%
0.454471	0.567473	5.148277255	4.5857	87.73%
0.578933	0.31082	4.896959318	4.876	99.57%
0.604808	0.040659	4.668580358	4.4587	95.29%
0.646044	0.24328	4.819004584	5.0309	95.79%

**Table 3:** Prediction of wear and comparison with actual wear (load: 392N).

LUMO energy	Total dipole	Prediction values	Actual values	Prediction rate
0.133606	0.216264	4.923744177	5.3027	92.85%
0.216042	0.081183	4.78432243	4.842	98.81%
0.411698	0.810617	5.349034102	4.8642	90.03%
0.454471	0.567473	5.129086755	4.4499	84.74%
0.578933	0.31082	4.873630555	4.8712	99.95%
0.604808	0.040659	4.635601811	4.4027	94.71%
0.646044	0.24328	4.79631669	4.9849	96.22%

Remarks: units of variables are: LUMO energy/Ha, Total dipole/D, Prediction values/mm, Actual values/mm

### Construction and importation of machine learning model between feature parameters and oxidation onset temperature

The oxidation onset temperature (OOT) data of the 17 groups of lubricants used for constructing the model are fundamentally taken from Shengpeng Zhan’s article^33^. Base oil used in the test has been ester oil TMPTO. Thermal oxidation test is carried out on a differential scanning calorimeter (DSC). The use of Tensorflow (Fig. 10) allows a linear regression machine learning model simulating characteristic parameters of lubricating oil and wear to be built. The characteristic parameters are basically including molecular energy, low orbital energy, LUMO-HOMO energy, dipole moment and fat-water partition coefficient. 13 groups of the parameters are nominated as training group and 4 other groups as prediction group. Data in individual sets are imported in Excle form, and subsequently their training relationship model is acquired post of many training iterations. The model is generally invoked via its database platform, and thereafter the prediction group is substituted to the model for predicting initial oxidation temperature. Analysis is therefore initiated by the predicted initial oxidation temperature which is then compared with its actually experimental values, and their predicted accuracy can be seen from Table 4.

**Table 4:** Prediction of oxidation one temperature and comparison with actual values.

Total energy	LUMO energy	LU-HO energy	Total dipole	AlogP	Prediction values	Actual values	Prediction rate
0.94444	0.929255	0.94843	0.043493	0.125003	224.31415	212.6	94.49%
0	0.921801	0.87674	0.056258	1	267.6886	254.5	94.82%
0.588314	0.99450	0.684217	0.368816	0.260944	264.62003	265.7	99.59%
0.888874	0.955542	0.944008	0.098562	0.18505	228.20561	228.7	99.78%

Remarks: units of variables are: Total energy, LUMO energy, LU-HO energy /Ha, Total dipole/D, Prediction values/°C, Actual values/°C.

## Conclusion

Lubrication is a core technology to support the advanced manufacturing, to ensure smooth operation of machinery, and to achieve energy saving. However, its process in developing the technology for accomplishment of high-performance lubricating material still relatively slow. It thus needs urgent innovation and concept in improving material design revolutionarily so that its practical significance can be achievable in developing efficient lubricating materials.

This paper is initiated on the basis of research idea on material genetic engineering for carrying out performance prediction of materials so as to meet extreme service performance. To accomplishing such purpose, technique in producing software of database for integrating the simulation calculation and experimental data, composition of the lubrication material-structure and physical parameter-lubrication performance is thus proposed. The technique combines database, data mining and other machine learning methods together. The anticipated database platform for lubricating materials has initially established by considering the following factors and components.

1. A database platform with high throughput computing results and test counterparts, and high capability to store and analyze the relevant data has thus been constructed based on Web, which can effectively realize data entry, data query and performance prediction of lubricating materials.
2. Software combining database with machine learning, the relationship model between calculated physical parameters and properties of lubricating materials has been established to facilitate the prediction of material properties.
3. The models of calculated physical parameters and wear rate, calculated physical parameters and oxidation onset temperature of lubricating oils were also constructed for predicting tribological properties and anti-oxidative properties of lubricating oils. Comparison of experimental results with the data predicted from model has shown its high level of conformability between prediction and test data.
